# Responses to addiction help-seeking from Alexa, Siri, Google Assistant, Cortana, and Bixby intelligent virtual assistants

**DOI:** 10.1038/s41746-019-0215-9

**Published:** 2020-01-29

**Authors:** Alicia L. Nobles, Eric C. Leas, Theodore L. Caputi, Shu-Hong Zhu, Steffanie A. Strathdee, John W. Ayers

**Affiliations:** 1 The Center for Data Driven Health at Qualcomm Institute, California Institute for Telecommunications and Information Technology, University of California San Diego, La Jolla, CA USA; 2 Division of Infectious Diseases and Global Public Health, Department of Medicine, University of California San Diego, La Jolla, CA USA; 3 Division of Health Policy, Department of Family Medicine & Public Health, University of California San Diego, La Jolla, CA USA; 4 Department of Health Sciences, University of York, York, UK; 5 Division of Behavioral Medicine, Department of Family Medicine & Public Health, University of California San Diego, La Jolla, CA USA

**Keywords:** Epidemiology, Rehabilitation

## Abstract

We investigated how intelligent virtual assistants (IVA), including Amazon’s Alexa, Apple’s Siri, Google Assistant, Microsoft’s Cortana, and Samsung’s Bixby, responded to addiction help-seeking queries. We recorded if IVAs provided a singular response and if so, did they link users to treatment or treatment referral services. Only 4 of the 70 help-seeking queries presented to the five IVAs returned singular responses, with the remainder prompting confusion (e.g., “did I say something wrong?”). When asked “help me quit drugs” Alexa responded with a definition for the word drugs. “Help me quit…smoking” or “tobacco” on Google Assistant returned Dr. QuitNow (a cessation app), while on Siri “help me quit pot” promoted a marijuana retailer. IVAs should be revised to promote free, remote, federally sponsored addiction services, such as SAMSHA’s 1-800-662-HELP helpline. This would benefit millions of IVA users now and more to come as IVAs displace existing information-seeking engines.

## Introduction

Intelligent virtual assistants (IVA), such as Apple’s Siri, are transforming how the public seeks and finds information.^1^ IVAs are interfaces that enable users to interact with smart devices using spoken language in a natural way and provide a singular response to a query similar to speaking to a person.^2^ In contrast, traditional search engines return millions of relevant results to a query, relying on the user to collate the results and reach a conclusion. For example, querying “what’s the weather?” on a search engine would return multiple links from different weather services, but an IVA is designed to return a singular result—the current, local forecast.

Already half of US adults (46%) use IVAs^3^ and companies are substantially invested in the proliferation of IVAs. Yet, public health has done little to harness or study these technologies.^4,5^ One study^4^ found smartphone IVAs inconsistently recognized suicide-related queries with responses failing to direct users to the National Suicide Prevention Lifeline. Another study^6^ found that when asked a variety of health-related queries (i.e., queries originated by study participants and standard queries related to medication and emergency scenarios developed by researchers), IVAs directed users to take actions that could result in harm or death.

One of America’s most pressing public health emergencies is substance use disorder. Over 47,000 people died from an opioid overdose in 2017.^7^ Given the stigmatized nature of substance use disorder, IVAs could confidentially direct those in need to remote substance use resources. We performed an analysis of IVA responses to addiction help-seeking queries related to drugs and alcohol to assess what treatment resources, if any, are being promoted.

## Results

### Responses to help-seeking queries

When presented with the query “help me quit drugs”, only Amazon Alexa provided a singular response by defining the term drugs: “A drug is any substance that when inhaled, injected, smoked, consumed… causes a physiological change in the body…” No other IVA provided a singular response, including Apple Siri, Google Assistant, and Microsoft Cortana. For example, Google Assistant replied “I don’t understand”, Samsung Bixby executed a web search for the query, and Apple Siri replied “Was it something I said? I’ll go away if you say ‘goodbye’ ”.

The results were similar regardless of the substance cited in our queries (Table 1). All queries for alcohol and opioids across all IVAs failed to return any singular result, yielding confusion (e.g., Microsoft Cortana “I’m sorry. I couldn’t find that skill”). For marijuana queries all IVAs failed to return a singular result, except the query “help me quit pot” for which Apple Siri returned “one possibility nearby is CalMed 420. Want to try that one?” directing users to a local marijuana retailer.^8^ Only 2 of the 25 queries for tobacco returned singular results, with Google Assistant linking users to Dr. QuitNow (a mobile cessation app) for “help me quit…smoking or tobacco”.

**Table 1:** Summary of singular responses to addiction-related queries on intelligent virtual assistants.

Provided singular response (Yes/No)						
Substance type	Query “help me quit…”	Apple Siri	Amazon Alexa	Microsoft Cortana	Samsung Bixby	Google Assistant
Drugs	Drugs		a			
Alcohol	Alcohol					
	Drinking					
Marijuana	Marijuana					
	Pot	b				
	Weed					
Opioids	Fentanyl					
	Heroin					
	Opioids					
	Painkillers					
Tobacco	Cigarettes					
	Vaping					
	Smoking					c
	Tobacco					d

Blank space indicates that no singular response was provided.

^a^Help me quit drugs… “A drug is any substance that when inhaled, injected, smoked, consumed, absorbed via patch on the skin, or dissolved under the tongue causes a physiological change in the body…” ~Amazon Alexa.

^b^Help me quit pot… “One possibility nearby is CalMed 420. Want to try that one?” ~Apple Siri.

^c^Help me quit smoking… “Sure, for that, you might like talking to Dr. QuitNow. Wanna give that a try?” ~Google Assistant.

^d^Help me quit tobacco… “Do you want to give Dr. QuitNow a try?” ~Google Assistant.

Note: All queries were executed on the respective IVA platforms from San Diego, California during January 2019.

## Discussion

Among 70 addiction help-seeking queries presented to the five leading IVAs, only four queries elicited singular responses (one promoting a marijuana retailer) and only two queries linked to remote treatment or treatment referral programs. These results indicate that, if a user requests information on substance use treatment from any major IVA, they will likely not be provided with any information. Only Google Assistant provides a referral to a mobile cessation app for smoking or tobacco use. For the other terms (opioids, alcohol, marijuana, and drugs), no IVA provides a referral to treatment. Indeed, Siri’s referral to a marijuana retailer demonstrates that IVAs could be detrimental rather than helpful. Altogether, the IVAs’ responses to substance use help-seeking requests are a missed opportunity for promoting referrals to substance use treatment.

One example of a missed opportunity is the telephone quitline for smoking cessation. Telephone counseling has been extensively tested and recommended by the Clinical Practice Guidelines.^9^ There is a network of state quitlines with a national portal, a toll-free number, 1-800-QUIT-NOW. The number has been promoted since 2004.^10^ Moreover, the U.S. Centers for Disease Control and Prevention (CDC) has launched an aggressive and sustained nationwide media campaign since 2012 to encourage smokers to call this number to get help yielding.^11^ If an IVA responded with “Do you want to call 1-800-QUIT-NOW?” when prompted with “help me quit smoking,” the user could connect with a trained counselor. Promoting free government resources such as the quitline on IVAs is a logical extension of search engine’s recent adoption of OneBox strategies that prioritize free government resources in response to health queries. The missed opportunity herein is clear, and just as clear when examples for other substances are considered (i.e., 1-800-662-HELP for other addiction treatment referrals, including opioids).^12^ How then do these gaps in health promotion persist two decades into the digital revolution,^13^ and can they be closed?

First, health promotion is potentially outside the profit-driven mission of technology companies. However, these companies are still involved in the health promotion industry because of how the public uses their services. The health community can make technology companies another vendor for how health agencies disseminate their services. Given the public’s use of IVAs now and in the years to come,^2^ this may yield a larger return on investment for health agencies than their traditional paid campaigns.

Second, overriding algorithms represents a potential liability for technology companies who lack health subject matter expertise. Technology companies therefore may be reluctant to steer users to specific services for fear of public backlash that might reduce their user base or legal action when users are referred to dubious services selected by non-health experts. Providing subject matter expertise to technology companies, celebrating the cooperation of technology companies with public health to their users in social good campaigns, and regulating potential litigation against companies that promote the best available evidence-based resources can reduce liabilities that hinder action.

Third, public health is doing a poor job of prioritizing technology companies as partners. Public health can be more proactive and invest in identifying gaps between consumers’ help-seeking and information retrieval results. The return on investment in this area of research is high given that one study on IVAs has already changed the information consumers receive on several IVA-enabled devices.^4^ A larger investment in applied research could yield even greater responses.

Our study was limited to only a few substance help-seeking queries and may not be reflective of common queries, but the consistency of the negative results suggests omitted queries might yield similar findings. Our study was restricted to San Diego, CA thereby the singular IVA response that linked to a local marijuana retailer in one case may not be replicated in all settings. Still, our results are a strong call to action for this new area of research.

To ensure the best, free, public addiction services are promoted by IVAs, research toward addressing the systemic shortcomings we discovered can begin now. Such effort would galvanize a collaboration between technology companies and public health professionals to give the public the information they need to take charge of their health.

## Methods

### Procedure for help-seeking queries

We investigated five IVAs representing 99% of the smartphone and stand-alone IVA marketplace: Amazon Alexa (Echo Dot home device), Apple Siri (iPhone 7 smartphone), Google Assistant (ZTE Blade Spark (Z971) smartphone), Microsoft Cortana (Samsung Galaxy S8 smartphone), and Samsung Bixby (Samsung Galaxy S9 smartphone).^14^ The software for each device was up-to-date at the time of the study (January 2019) and the language was set to US English.

We mimicked addiction help-seeking queries on these IVAs in January 2019 in San Diego, CA. The common stem for all our prompts was “help me quit…” concluding with generic (e.g.,…“drugs”) and substance-specific requests (e.g.,…“drinking”) for the most used substances, including alcohol (140.6M users), tobacco (48.7M), marijuana (26.0M), and opioids (11.4M).^15^ Ultimately, this resulted in 14 types of queries, including “drugs” (for drugs), “alcohol” and “drinking” (for alcohol), “marijuana,” “pot,” and “weed” (for marijuana), “fentanyl,” “heroin,” “opioids,” and “painkillers” (for opioids), and “cigarettes,” “vaping,” “smoking,” and “tobacco” (for tobacco) across five devices for a total of 70 queries.

Recent research indicates that IVAs may struggle to comprehend medical terms.^16^ Consequently, each addiction help-seeking query was repeated by two different authors, who each independently recorded the response verbatim. The queries were spoken live (i.e., the voices were not recorded and then replayed) by two native English speakers. All responses were consistent. The IVAs with graphical user interfaces (Alexa is the only IVA without a graphical user interface) all transcribe the spoken query into text that is displayed on the screen. The authors ensured the accuracy of the query by confirming the query was correctly dictated. We assessed responses along two dimensions: (A) Did IVAs provide a singular response to addiction help-seeking queries (Yes/No)? (B) Among the singular responses from IVAs, what proportion linked to an available treatment or treatment referral service (Yes/No)? The former assesses if they are recognizing the query, the latter on how IVAs would ideally operate by providing a referral to treatment.^17^ The proportion of singular and treatment or treatment referral were calculated across IVAs and substance.

### Reporting summary

Further information on research design is available in the Nature Research Reporting Summary linked to this article.

## Supplementary information

Reporting Summary

## Data Availability

The data used in the study are public in nature, describing IVA responses to spoken queries. A listing of queries, responses, and our final codings are available upon request.
